# The novel high-affinity humanized antibody IMM40H targets CD70, eliminates tumors via Fc-mediated effector functions, and interrupts CD70/CD27 signaling

**DOI:** 10.3389/fonc.2023.1240061

**Published:** 2023-10-02

**Authors:** Song Li, Dianze Chen, Huiqin Guo, Dandan Liu, Chunmei Yang, Ruliang Zhang, Tianxiang Wang, Fan Zhang, Xing Bai, Yanan Yang, Nana Sun, Wei Zhang, Li Zhang, Gui Zhao, Liang Peng, Xiaoping Tu, Wenzhi Tian

**Affiliations:** ^1^ Department of R&D, ImmuneOnco Biopharmaceuticals (Shanghai) Inc., Shanghai, China; ^2^ Department of CMC, ImmuneOnco Biopharmaceuticals (Shanghai) Inc., Shanghai, China

**Keywords:** IMM40H, CD70/CD27 signaling, CD70, targeting CD70 antibody, ADCC

## Abstract

**Background:**

A significant level of CD70 can be detected in various types of tumor tissues and CD27 is expressed on Treg cells, but CD70 expression is low in normal tissues. The interaction between CD70 and CD27 can stimulate the proliferation and survival of cancer cells and increase the level of soluble CD27, which is associated with poor prognosis in patients with lymphoma and certain solid tumors. Thus, it is a promising therapeutic target for the treatment of many major CD70+ cancer indications, including CD70+ lymphoma, RCC, NSCLC, HNSCC and OC.

**Methods:**

IMM40H was obtained through hybridoma screening and antibody humanization techniques. IMM40H was evaluated for its binding, blocking, Fc-dependent effector functions and antitumor activity characteristics in various *in vitro* and *in vivo* systems. The safety and tolerability profile of IMM40H were evaluated through single and repeated administration in cynomolgus monkeys.

**Results:**

*In vitro* cell-based assays demonstrated that IMM40H had considerably stronger CD70-binding affinity than competitor anti-CD70 antibodies, including cusatuzumab, which enabled it to block the interaction of between CD70 and CD27 more effectively. IMM40H also exhibited potent Fc-dependent effector functions (ADCC/CDC/ADCP), and could make a strong immune attack on tumor cells and enhance therapeutic efficacy. Preclinical findings showed that IMM40H had potent antitumor activity in multiple myeloma U266B1 xenograft model, and could eradicate subcutaneously established tumors at a low dose of 0.3 mg/kg. IMM40H (0.3 mg/kg) showed therapeutic effects faster than cusatuzumab (1 mg/kg). A strong synergistic effect between IMM01 (SIRPα-Fc fusion protein) and IMM40H was recorded in Burkitt’s lymphoma Raji and renal carcinoma cell A498 tumor models. In cynomolgus monkeys, the highest non-severely toxic dose (HNSTD) for repeat-dose toxicity was up to 30 mg/kg, while the maximum tolerated dose (MTD) for single-dose toxicity was up to 100 mg/kg, confirming that IMM40H had a good safety and tolerability profile.

**Conclusion:**

IMM40H is a high-affinity humanized IgG1 specifically targeting the CD70 monoclonal antibody with enhanced Fc-dependent activities. IMM40H has a dual mechanism of action: inducing cytotoxicity against CD70+ tumor cells via various effector functions (ADCC, ADCP and CDC) and obstructs the proliferation and activation of Tregs by inhibiting CD70/CD27 signaling.

## 1 Introduction

Cluster of differentiation 70 (CD70) is a tumor necrosis factor family cell surface antigen. It is involved in lymphocyte maturation and proliferation and is intermittently produced by mature dendritic cells and a small proportion of activated B and T lymphocytes (1, 2). However, solid and hematological cancers express CD70 constitutively, and this expression is associated with a poor prognosis (3–6). Trimer type II transmembrane protein CD70 is the most common version of CD70, while CD27 is its receptor. The secretion of soluble CD27 (sCD27) and proteolytic shedding of the ectodomain of CD27 occur after CD70 binds to CD27 (CD70-CD27), which activates the nuclear factor-κB (NF-κB) and c-Jun kinase pathways and promotes the proliferation and survival of malignant cells (7). The CD70-CD27 signaling pathway can promote regulatory T cells (Tregs) mobilization or survival, leading to immune monitoring in the tumor microenvironment (8).

Monoclonal antibodies are the main focus of CD70-targeted immunotherapy, and they have either been tested as monotherapeutic agents or in conjunction with other medications. CD70 antibodies are not available commercially. For inhibiting CD70/CD27 signaling, cusatuzumab (ARGX-110) is the most rapidly progressing anti-CD70 monoclonal antibody (9). Due to its effector actions, such as antibody-dependent cellular phagocytosis (ADCP), complement-dependent cytotoxicity (CDC), and increased antibody-dependent cell-mediated cytotoxicity (ADCC), cusatuzumab can destroy tumor cells directly (9). Combination treatment with azacitidine and cusatuzumab is safe and effective for individuals with untreated AML who are not candidates for intense chemotherapy, as determined by the results of a Phase I/II trial. Cusatuzumab may cause long-lasting remissions by eliminating CD70+ leukemia stem cells (LSCs) (10–12).

In this study, we reported the novel targeting CD70 monoclonal antibody IMM40H, which has higher affinity and stronger blocking activity than competitor anti-CD70 antibodies. Besides enhancing the immune defense against tumors by disrupting the communication between CD70-CD27 and Tregs, IMM40H induces cytotoxicity over CD70+ tumor cells through several effector functions (ADCC, ADCP, and CDC). Preclinical data have shown that IMM40H has potent antitumor activity in various CDX models, including Raji, U266B1, and A498 tumor cells; IMM40H was also safe and well-tolerated in cynomolgus monkeys.

## 2 Materials and methods

### 2.1 Cell culture

The SP2/0, Raji, Daudi, Jurkat, and Jeko-1 cell lines were purchased from the Cell Bank of the Chinese Academy of Sciences, and the U266B1 and A498 cell lines were purchased from the American Type Culture Collection (ATCC). Jurkat-CAR-CD27 and FcgRIIIA (158V) target-activated NK (FcR-TANK™) cells were self-modified in our laboratory. The logarithmic growth phase was reached in all cell lines before harvesting. The SP2/0, Raji, U266B1, Daudi, Jurkat, Jurkat-CAR-CD27, and Jeko-1 cell lines were maintained in an incubator at 37°C and 5% CO_2_ with the RPMI-1640 medium (Gibco, Cat#11875093) containing 10% fetal bovine serum (Gibco, Cat#10091148) and 1% penicillin-streptomycin (Gibco, Cat#15140122). MEM medium (Gibco, Cat#11095080) with 1% penicillin-streptomycin and 10% FBS was used for the cultivation of A498 cells. TANK serum-free medium (Immuneonco, Cat#CT001-1) was used for the cultivation of FcR-TANK cells.

### 2.2 Humanization and development of antibodies

After immunizing with the human CD70 full-length extracellular domain (39–193) fused to mIgG1-Fc, we used the conventional hybridoma technique to screen for anti-human CD70 antibodies. Positive fusions were evaluated for specific CD70 binding to U266B1 by fusing splenocytes from vaccinated animals with the Sp2/0 myeloma cell line. We cloned and sequenced one of the hybridoma clones, designated 26A3. By grafting CDRs onto human germline frameworks, 26A3 was humanized. For detailed information on the preparation of IMM40H, can refer to the approved patent (US11613584B1).

### 2.3 Antibody expression, purification, and characterization

CHO-S cells were cultured in TransFx-C CHO Transient transfection medium (Hyclone, Cat#SH30942.02). Co-transfection of expression vectors expressing the antibody heavy chain and light chain was achieved by transient transfection using a polyethylenimine transfection reagent (Polysciences, Cat#24765). After 8–10 days, the cells were collected for their supernatants and loaded to Protein A Sepharose columns (Bestchrom, Cat#AA0273). Wash buffer (20 mM phosphate buffer (PB) +140 mM NaCl, pH7.4 ± 0.1) was added to the columns, and the antibodies were then eluted with the elution buffer (25 mM NaAc+ 100 mM NaCl, pH3.5 ± 0.1). Using 2 M Tris, the pH of the collected fractions was adjusted to 5.2 ± 0.2. Size-exclusion high-performance liquid chromatography (SEC-HPLC) was performed to examine the purity of the eluted antibodies.

### 2.4 Determining the binding of IMM40H to the trimer CD70 protein

The binding of IMM40H to the trimer CD70 protein (ACROBiosystems, Cat# CDL-H52Da) was analyzed by ELISA. Trimer CD70 protein (100 ng/well) in PBS was overnight incubated at 4°C in flat-bottom 96-well plates (Thermo Fisher Scientific, Cat#442404). The plates were blocked in blocking buffer (PBS + 3% skim milk) at room temperature for 2 h, then washed thrice with wash buffer (PBS + 0.05% Tween-20). The plates were incubated with two-fold serially diluted IMM40H at the starting concentration of 10 µg/mL. Peroxidase-conjugated anti-human IgG secondary antibody (Jackson Immuno, Cat#109–006–008) was added after washing thrice and incubated for 1 h. After incubating the plates with the substrate solution TMB (KPL, Cat#51200050) for 10 min and adding 2 M sulfuric acid to terminate the reaction, the plates were analyzed using a microplate reader (BioRad, iMARK).

### 2.5 Determining the binding of IMM40H to CD70+ tumor cells

The binding of IMM40H to CD70+ tumor cells (including Raji, U266B1, SP53, and A498) was analyzed via flow cytometry assays. We used hIgG1-Fc (In house) as an isotype control. The tumor cells were incubated with IMM40H, Cusatuzumab, and hIgG1-Fc at different serially diluted concentrations for 45 min at 4°C. Then, PBS containing 0.5% BSA (Sangon Biotech, Cat#A500023–0100) was added to remove free antibodies. The 500-fold diluted FITC-conjugated anti-human IgG Fc 2nd antibody (Sigma, Cat#F9512) was added to the samples and incubated at 4°C for 45 min in the dark. After washing, the FITC fluorescence signal of the cells was analyzed by performing flow cytometry assays (Luminex, Guava^®^ easyCyte™ 8HT Base System).

### 2.6 Antibody affinity assay

Surface plasmon resonance, SPR device (Biacore T200, GE Healthcare) was used for evaluating the affinity of IMM40H to recombinant human CD70 trimer. Following the amine coupling methodology described by the manufacturer, the anti-human IgG(Fc) antibody (GE Healthcare, Cat# BR-1008–39) was diluted to 25 µg/mL in 10 mM sodium acetate (pH 5.0) and then attached to a CM5 biosensor (GE Healthcare, Cat#BR100530). The SPR experiment was performed at 25°C in 1xHEPES running buffer (pH 7.4, 10 mM HEPES, 150 mM NaCl, 3 mM EDTA, and 0.005%Tween-20). The samples, which were diluted to 10 µg/mL in 1xHEPES (pH 7.4), were captured on the anti-hIgG(Fc) antibody surface. A concentration series of 3.125–0.049 nM trivalent human CD70 was injected over the captured antibodies at 30 µL/min to measure association and dissociation. The anti-hIgG(Fc) antibody capture surface was regenerated between test cycles with 30 s injections of 3 M magnesium chloride. Biacore T200 Evaluation Software v.3.1 was used to fit the rate constants ka (kon, association rate) and kd (koff, dissociation rate) from the reference flow cell and 0 nM blank-subtracted sensorgrams to a 1:1 binding model.

### 2.7 Blocking the CD70/CD27 signaling assay

We engineered a recombinant Jurkat cell line that expressed a chimeric CD27 receptor (Jurkat-CAR-CD27) to further define the target-blocking action in a biological setting. The chimeric antigen receptor (CAR)-CD27 consisted of an extracellular CD27 domain that was linked to CD8α-hinge, CD28-TMD/ICD, and CD3ζ signal domains in a specific order. When the Jurkat-CAR-CD27 cells were incubated with recombinant Raji cells (CD70+) for 24 h, the former cells underwent activation-induced cell death (AICD), which was coupled with an increase in the expression of CD69. However, when the interaction between CD27 and CD70 was blocked by CD70 mAb, CD70-induced cell death was inhibited and CD69 expression remained stable. The experimental steps are briefly described as follows. The Jurkat-CAR-CD27 cells and Raji cells were mixed in a 5:1 ratio and incubated with serially diluted concentrations of antibodies in a humidified incubator at 5% CO_2_ and 37°C for 24 hours. After incubation, CD69 (Biolegend, Cat#310906) and CD3 (Biolegend, Cat#300412) antibodies were added to each well, and then, the expression of CD69 on Jurkat-CAR-CD27 cells was determined via flow cytometry assays.

### 2.8 *In vitro* Fc-mediated effector function (ADCC/ADCP/CDC) assay

#### 2.8.1 ADCC assay

The target cells were labeled with 200 nM carboxyfluorescein succinimidyl ester (CSFE; Sigma, Cat#21888) and incubated with different concentrations of antibodies for 30 min. Then, in an E/T ratio of 1:2, FcgRIIIA (158V) target-activated NK (FcR-TANK™) cells (In house) were added to wells for 4 h at 5% CO_2_ and 37°C. Propidium Iodide (PI) solution (Sigma, Cat#P4170) was used for staining the cells. Then, the flow cytometry assay was performed to collect the cells, and the PI-positive stained cells were calculated. The intensity of ADCC was calculated using the formula: Lysis% = [(E+T+Ab) % PI positive cell – (E+T) % PI positive cell]/(100 – T % PI positive cell) × 100%.

#### 2.8.2 ADCP assay

We collected THP-1 cells and washed them in RPMI-1640 medium with 10% fetal bovine serum (Gibco, Cat# 10091148) and 1% penicillin-streptomycin (Gibco, Cat# 15140122). In a flat 96-well plate, 100 µL of 4 × 10^5^/mL THP-1 cells and 200 ng/mL PMA (Sigma, Cat#P-050) were incubated for 48 h at 37°C and 5% CO_2_. After counting and harvesting the target cells, 200 nM CSFE was added to the cells and incubated at 37°C in the dark for 30 min to label them. After washing twice with full culture medium, the target cells were seeded at a density of 1 × 10^5^ cells/well (50 µL, 2 × 10^6^/mL). Antibodies were serially diluted and added to the plate at 50 µL/well and incubated for 2 h (Effector : Target = 2:5) at 37°C and 5% CO_2_. After incubation, the plates were washed with PBS to remove free target cells. Adherent macrophages were digested with 10 µL/well 0.25% Trypsin-EDTA and resuspended in 150 µL/well PBS buffer. The phagocytic index was determined by flow cytometer and defined as the percentage of macrophages that have phagocytosed the target cells. The phagocytic index was calculated using the formula: phagocytic index % = (E+T+Ab) % CFSE positive cell – (E+T) % CFSE positive cell.

#### 2.8.3 CDC assay

Different concentrations of antibodies were used for incubating target cells with normal human serum complement (Quidel, Cat#A113) at 37°C and 5% CO_2_ for 4 h before being stained with a PI solution. A flow cytometry assay was performed to collect the cells and the percentage of PI-positive cells was calculated. The CDC intensity was calculated using the formula: Lysis % = Experimental Sample Lysis %- No Antibody Lysis %.

### 2.9 *In vivo* xenograft mouse model

#### 2.9.1 U266B1/A498 xenograft model in CB17-SCID mice

We resuspended 200 µL of cells in cold PBS and injected them into the right side of the back near the axilla of female CB17- severe combined immunodeficient (SCID) mice aged 6-8 weeks (5 × 10^6^ A498 or U266B1 cells). After the tumors in both models grew to an average of 126 and 195 mm^3^, respectively, the animals were randomly divided into treatment groups (eight mice per group) based on the size of the tumor and the weight of the mice. In both experiments, IMM40H was administered intraperitoneally. In the U266B1 study, 0.3, 1, and 3 mg/kg QW of IMM40H was administered for four weeks. Bortezomib (Xian Janssen) was IV administered at 0.5 mg/kg BIW for four weeks and Cusatuzumab was administered via IV at 1 mg/kg QW for four weeks. In the A498 study, 3, 10, 30 mg/kg IMM40H, 10 mg/kg IMM01, and 10 mg/kg IMM40H combo with 5 mg/kg SIRPα-Fc fusion protein IMM01 were administered BIW for four weeks. The tumor volume and body weight were measured twice a week. When the tumor size met the euthanasia threshold (3,000 mm^3^), the animals were euthanized. When a sufficient number of mice in any group met the euthanasia threshold, the study was terminated.

#### 2.9.2 Raji orthotopic xenograft model in CB17-SCID mice

To establish disseminated disease, female CB-17 SCID mice aged 6-8 weeks were injected with 100 µL, 5 × 10^6^ Raji cells resuspending cold PBS per animal into the lateral tail vein. The animals were randomized to various treatment groups (eight mice per group) based on body weight (BW) after three days of tumor cell inoculation. Treatment began with twice-weekly tail vein injections for three consecutive weeks. The following dosages were used: IMM40H (1, 3, and 10 mg/kg), IMM01 (0.3 mg/kg), and IMM40H (3 mg/kg) with IMM01 (0.3 mg/kg). The mice were euthanized when they displayed symptoms of an excessive tumor burden, such as weight loss > 20%, stooped posture, paralysis, lethargy, cranial edema, or dehydration. The BW of the mice was monitored at least twice a week.

### 2.10 Pharmacokinetics study in non-human primates

Acute toxicity, Pharmacokinetics (PK), and ToxicoKinetics (TK) profiles of IMM40H were evaluated following intravenous (IV) administration in cynomolgus monkeys. The appropriate standard operating procedures (SOPs) provided by WuXi AppTec (Suzhou) Co., Ltd. were followed for all experiments. The Protocol complied with the requirements of the Animal Welfare Act Regulations (9 CFR 3).

Acute toxicity study was evaluated after IMM40H was administered as a single-dose IV infusion to cynomolgus monkeys. Eight cynomolgus monkeys (4 animals/sex) were randomly assigned to four groups (1/sex/group). Different concentrations of IMM40H (0, 20, 50, and 100 mg/kg) were administered to the monkeys in different groups. The animals were monitored for 14 days and various parameters were assessed, including viability (morbidity/mortality), autopsy, clinical pathology and observations, food intake, and body weight.

The IMM40H PK study was conducted after a single IV infusion was administered to male and female cynomolgus monkeys to determine the serum PK properties and evaluating the presence of anti-drug antibodies (ADA) in serum. In total, 18 cynomolgus monkeys (9 animals/sex) were assigned to three groups (3 animals/sex/group), which were IV-administered with different concentrations of IMM40H (0.5, 1.5, or 3 mg/kg). Blood samples were collected from the peripheral vessel of the animals following administration after 0, 0.5, 1, 2, 8, 24, 48, 72, 96, 120, 168, 336, 504, and 672 h. ADA samples were collected following administration after 0, 168, 336, 504, and 672 h. The concentration of IMM40H in serum was measured by an ELISA method, and ADA was analyzed using an ECL method.

In the TK study, male and female cynomolgus monkeys were characterized after they were administered IMM40H via IV infusion for five doses (on days 1, 8, 15, 22, and 29). In total, 40 monkeys (5/sex/group) were randomly assigned into four groups (0, 3,10, and 30 mg/kg) in the TK study. On days 1 and 22, blood samples were collected for the TK analysis at 0, 0.5, 2, 6, 24, 48, 72, 96, and 168 h after the end of the infusion.

### 2.11 Statistical analysis

We used GraphPad Prism 8.0 (GraphPad Software, Inc.) for statistical analysis. For comparisons involving three or more groups, a one-way repeated ANOVA was performed with Holm-Sidak correction. T-tests were performed for comparisons involving two groups. All differences were considered to be statistically significant at P ≤ 0.05. In figures, asterisks denote statistical significance (*p<0.05; **p<0.01; ***p<0.001; ****p<0.0001).

## 3 Results

### 3.1 Humanization and generation of anti-CD70 monoclonal antibodies

**Figure 1:**
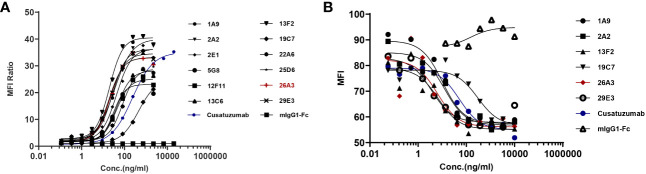
Identification of the lead anti-CD70 antibody. **(A)** Binding of the candidates to U266B1 cells, as determined by flow cytometry assays. **(B)** Blocking the interaction of CD27 with U266B1 cells, as determined by flow cytometry assays. Candidates with U266B1 cells were pre-incubated. Then, biotinylated-CD27 protein (Acro Biosystems, Cat#TN7-H82F6) and PE-conjugated Streptavidin (Biolegend, Cat# 405204) were added to detect the binding signal of CD27 and U266B1 cells. The results showed that 26A3 lead had the optimal binding and blocking activities, and thus, it was selected as the final candidate for generation.

The binding activity of the selected hybridoma clones to U266B1 cells was evaluated via FACS. One of the most promising positive clones, 26A3, could inhibit the interaction between human CD70 and CD27 in addition to binding human CD70 (**Figures 1A, B**).

**Figure 2:**
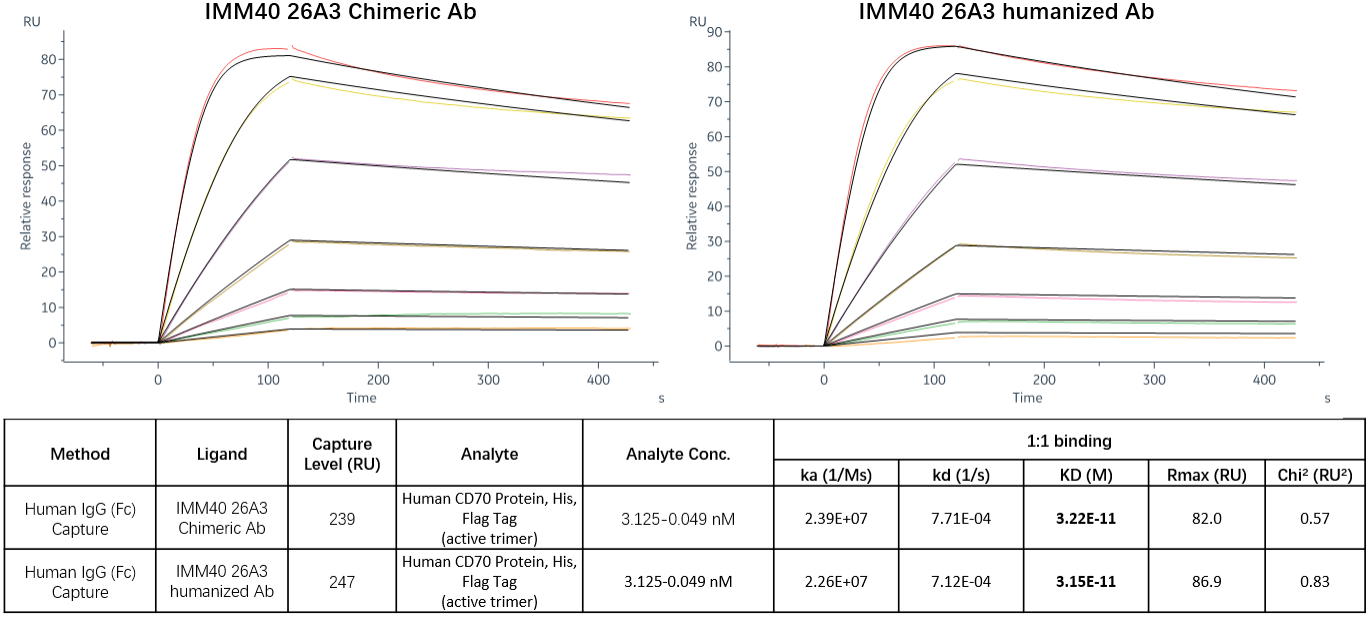
CD70 target affinity measured by SPR. The results of the affinity analysis showed that the humanized 26A3 (IMM40H) has equivalent CD70 binding affinity to the chimeric 26A3 (kD = 3.22E-11M for humanized Ab, kD = 3.15E-11M for chimeric Ab).

To improve its Fc-dependent effector capabilities, 26A3 was humanized by grafting its CDRs onto human germline frameworks, and then, it was built as human IgG1 with S298A, E333A, and K334A alterations. FACS, ELISA, and SPR were then used to evaluate the humanized 26A3 (IMM40H) for its antigen-binding affinity. The FACS and ELISA results showed that the humanized 26A3 antibody (IMM40H) had equivalent CD70-binding ability to the chimeric antibody (**Supplementary Figures 1A, B**). The results of the affinity analysis showed that IMM40H also had a similar CD70 binding affinity to that of the chimeric version (kD = 3.22E-11M for humanized Ab, kD = 3.15E-11M for chimeric Ab) (**Figure 2**). Also, 26A3 could cross-react with CD70 from cynomolgus monkey but not from mouse (**Supplementary Figure 2**).

### 3.2 IMM40H exhibited the strongest specific binding activity to CD70+ tumor cells

**Figure 3:**
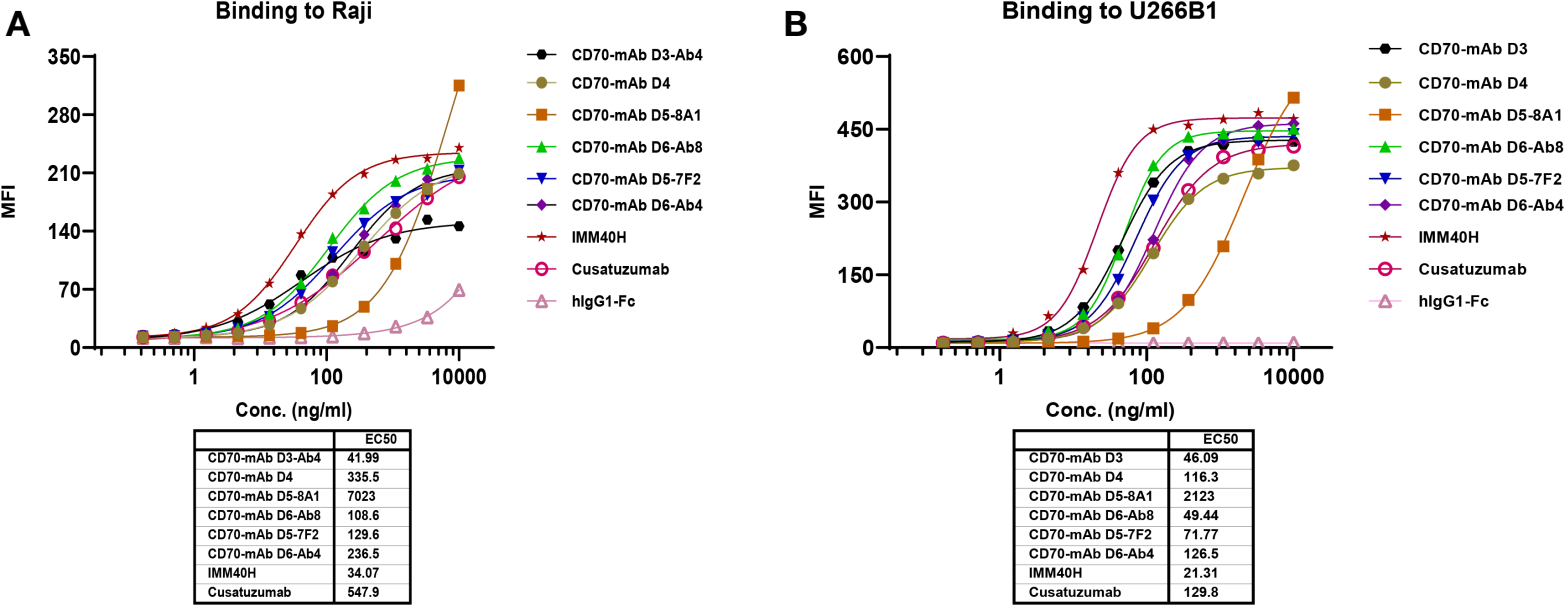
The binding of IMM40H and competing CD70 mAbs to CD70+ tumor cells was evaluated by FACS. **(A)** The binding of IMM40H and competing CD70 mAbs to Burkitt’s lymphoma Raji cell. **(B)** The binding of IMM40H and competing CD70 mAbs to multiple myeloma U266B1 cells. The results showed that IMM40H had a higher binding activity to Raji and U266B1 cells than the competitor anti-CD70 antibodies. Competitor anti-CD70 antibodies sequence derived from patents data. Cusatuzumab, WO2012123586A1; CD70-mAb D3, US20120294863A1; CD70-mAb D4, WO2007038637A2; CD70-mAb D5–8A1&CD70-mAb D5–7F2, WO2013192360A1; CD70-mAb D6-Ab4&CD70-mAb D6-Ab8, WO2013043933A2.

Only stromal cells from the thymic medulla and mature dendritic cells were found to contain CD70 protein, indicating that its distribution outside the lymphoid organs is quite restricted (13, 14). CD70 is extensively expressed in Hodgkin and non-Hodgkin lymphomas, chronic lymphocytic leukemia, and multiple myeloma. It is also substantially expressed in renal cells (15, 16). A therapeutic anti-CD70 antibody may have broader applications since aberrant CD70 expression is associated with a poor prognosis in solid and hematologic cancers. FACS analysis was used to determine whether IMM40H bound to CD70 on various solid and hematologic tumor cells. Subsequently, IMM40H showed the highest binding activity to Raji and U266B1 cells among the competitor anti-CD70 antibodies (**Figures 3A, B**). In the low nanomolar range, IMM40H bound to both A498 and SP53 cells with high affinity (**Supplementary Figures 3A-E**). Upon transfection with CD70, IMM40H bound to the CD70-negative cell line CHO (Chinese Hamster Ovary) and Jurkat (T cell lymphoma cell line), proving its specificity for CD70 (**Supplementary Figures 4A, B**).

### 3.3 IMM40H exhibited the strongest blocking activity of interrupting CD70/CD27 signaling

**Figure 4:**
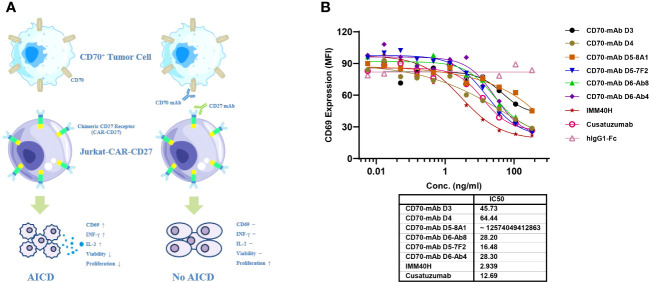
The target-blocking activity of IMM40H was characterized by bioassay using the Jurkat-CAR-CD27 cell line. **(A)** MOA of Jurkat-CAR-CD27 cell. Upon ligation with CD70+ positive tumor cell, the CAR-CD27 (extracellular CD27 domain that was linked to CD8α-hinge, CD28-TMD/ICD, and CD3ζ signal domains in a specific order) molecule transduces a stimulatory signal and activates Jurkat-CAR-CD27 cells. The activated Jurkat-CAR-CD27 cells up-regulate Fas and Fas ligand, upon interaction of Fas on one cell with Fas ligand on another cell, death signal will be transduced to Jurkat-CAR-CD27 cells, thus will undergo AICD. Which was coupled with an increase in the expression of CD69, INF-γ and IL-2. Antibodies specific for CD70 or CD27 can block the interaction of CD70 with extracellular CD27 domain and thus will block the CD70 induced AICD. **(B)** The target blocking activity of IMM40H was significantly better than that of the competing CD70 mAbs.

Inhibition of the CD70-CD27 interaction between tumor cells and immune cells in the tumor microenvironment can be used for therapeutic purposes. The expression of CD70 on cancer cells is linked to the induction of regulatory T cells, which may inhibit the immune system in the tumor microenvironment (17). Additionally, IMM40H can inhibit growth signals and/or limit the acquisition of T-cell immune regulatory function inside the tumor microenvironment, while removing CD70+ malignant cells through Fc-mediated effector activities. A chimeric CD27 receptor (CAR) expressing recombinant Jurkat cells (Jurkat-CAR-CD27) was tested in a bioassay to determine their target-blocking activity (**Figure 4A**). When Jurkat-CAR-CD27 bound to Raji (CD70+), it transduced a stimulatory signal, which upregulated the expression of CD69. IMM40H can block CD70-CD27 interaction, which in turn inhibits the expression of CD69. IMM40H has potent target-blocking activity (IC50 = 2.94 ng/mL), which is significantly better than that of cusatuzumab (IC50 = 12.69 ng/mL) and other competing CD70 mAbs (**Figure 4B**).

### 3.4 IMM40H exhibited potent antitumor effects through Fc-dependent effector functions *in vitro*

**Figure 5:**
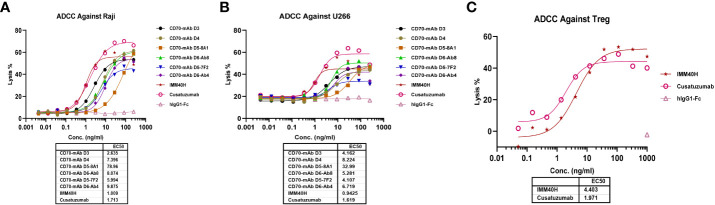
The ADCC activity against CD70+ tumor cells and Tregs was measured using FcR-TANK. **(A)** IMM40H and the competing CD70 mAbs induced ADCC against Raji. **(B)** IMM40H and competing CD70 mAbs induced ADCC against U266. **(C)** IMM40H induced ADCC against Tregs. The ADCC-inducing activity of IMM40H was stronger than the competing CD70 mAbs and had similar activity to defucosylated cusatuzumab.

**Figure 6:**
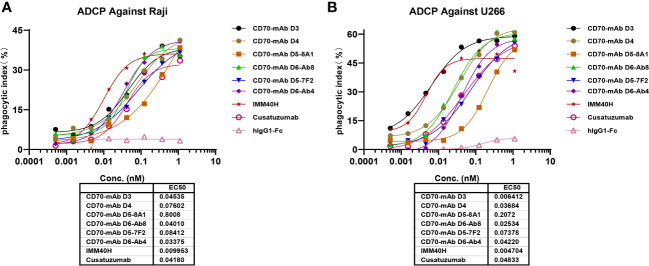
The ADCP activity against CD70+ tumor cells were measured using THP-1 cell. **(A)** IMM40H induced ADCP against Raji. **(B)** IMM40H induced ADCP against U266. The phagocytic index was determined by FACS and defined as the percentage of CFSE positive macrophages. The phagocytic index was calculated using the formula: phagocytic index % = (E+T+Ab) % CFSE positive cell – (E+T) % CFSE positive cell. IMM40H induced stronger ADCP against CD70+ tumor cells than the competing CD70 mAbs, including cusatuzumab.

In healthy tissues and organs, expression of the CD70 protein is very low. IgG1 was prioritized while developing anti-CD70 therapeutic antibodies since it has a high affinity for binding and activating FcγRs and can elicit potent ADCC and ADCP against CD70+ tumor cells. IgG1 was selected for the formats of IMM40H, with the S298A, E333A, and K334A substitution in the Fc region to enhance the Fc-dependent effector functions. *In vitro* pharmacology studies showed that IMM40H can induce tumor cell lysis through ADCC, ADCP, and CDC in malignant cells expressing CD70. IMM40H exhibited stronger ADCC activity against cells associated with hematological malignancies (Raji, U266B1, Daudi, and Jeko-1 cell lines) compared to competing CD70 mAbs, while also exhibiting potent killing effects against solid tumor cells (A498) (**Figures 5A, B**, **Supplementary Figures 5A-C**). A significant proportion of human Tregs gain stable CD70 expression while losing CD27 after prolonged *in vitro* stimulation (18). Using commercially activated Tregs (Sailybio, Cat#XFB-nTreg-02BA) as target cells, the results showed that due to the low expression of CD70 in Treg cells, IMM40H was able to directly kill Tregs through ADCC (**Figure 5C**, **Supplementary Figure 6**). Besides enhancing the immune defense against tumors by disrupting the communication between CD70-CD27 and Tregs, IMM40H can reduce the ratio of activated Tregs and relieve immune suppression by directly killing them. Using flow cytometry, phagocytosis was evaluated in the monocytic cell line THP-1 (effector) against the target cell lines Raji, Daudi, U266B1, Jeko-1, and A498 labeled with CFSE by measuring the THP-1 green fluorescence following 2 h of incubation with IMM40H. IMM40H induced stronger ADCP against Raji and U266B1 than the competing CD70 mAbs, including cusatuzumab (**Figures 6A, B**). ADCP effects on THP-1 cells with an EC_50_ range of MFI of 0.035 nM in Raji, 0.0094 nM in U266B1, 0.0718 nM in Daudi, 0.0052 nM in Jeko-1, and 0.0508 nM in A498, respectively (**Supplementary Figure 7**). Cell lysis as a marker for CDC was determined via propidium iodide (PI) labeling by flow cytometry assays. The lysis EC_50_ for IMM40H was 0.395 nM in Raji cells (**Supplementary Figure 8**), whereas IMM40H had no CDC activity against other CD70+ tumor cell lines, including U266B1, Daudi, Jeko-1, and A498 (data not presented).

### 3.5 IMM40H exhibited strong antitumor efficacy *in vivo*

#### 3.5.1 *In vivo* activity against multiple myeloma (U266B1) subcutaneous xenograft model

**Figure 7:**
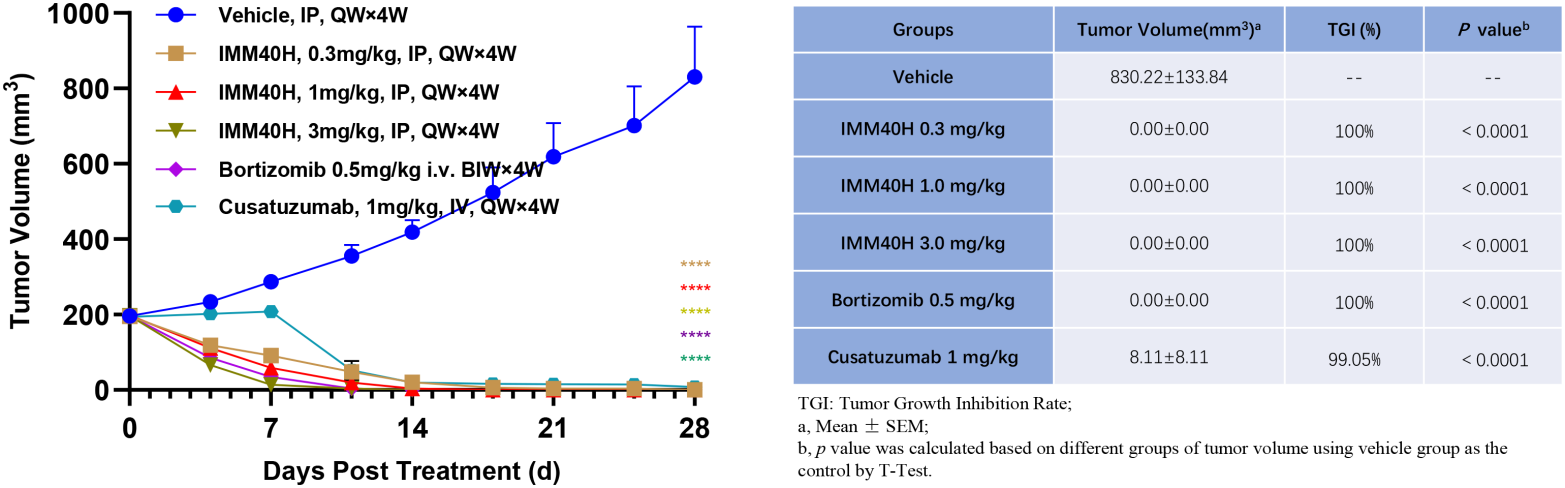
*In vivo* activity against multiple myeloma (U266B1) subcutaneous xenograft model. IMM40H demonstrates much stronger tumor killing efficacy than Cusatuzumab. Therapeutic effect was observed earlier in IMM40H (0.3 mg/kg) group than in cusatuzumab (1 mg/kg) group.

We examined the effect of IMM40H in subcutaneous xenograft models derived from the multiple myeloma cell line U266B1 compared to the effects of bortezomib and Cusatuzumab, which are commonly used for multiple myeloma treatment. IMM40H (IP, QW×4, 0.3, 1, 3 mg/kg), Bortezomib (IV, BIW×4W, 0.5 mg/kg), and Cusatuzumab (IV, QW×4, 1 mg/kg) were administered after grouping. Animals in all groups showed no significant abnormality during the study, without any remarkable body weight differences among animals in the 0.3, 1, and 3 mg/kg IMM40H groups. At the endpoint of the study, all mouse tumors in the three IMM40H treatment groups were eliminated. The therapeutic effect was observed earlier with IMM40H (0.3 mg/kg) than with cusatuzumab (1 mg/kg) (**Figure 7**).

#### 3.5.2 *In vivo* activity against Burkitt’s lymphoma (Raji) orthotopic xenograft model

**Figure 8:**
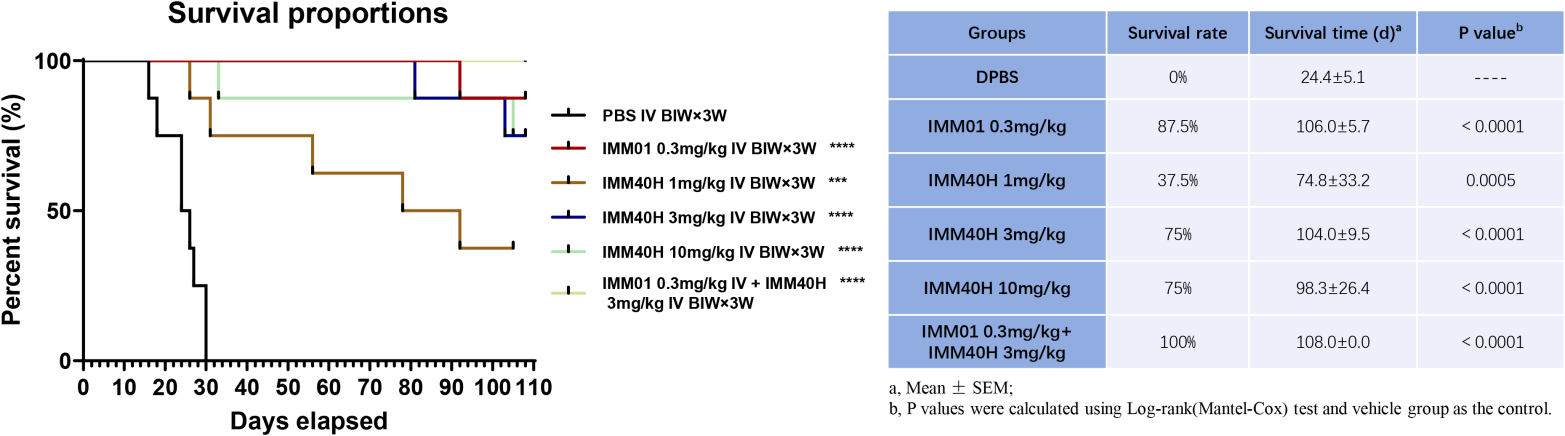
*In vivo* activity against Burkitt’s lymphoma (Raji) orthotopic xenograft model. IMM40H substantially improved the survival time in a dose-dependent manner. The percentage of mice survived in the three dosage group (1, 3, 10 mg/kg) is 38% (3/8), 75% (6/8), and 75% (6/8) respectively. Interestingly, a combination of IMM01 (0.3mg/kg) with IMM40H (3mg/kg) showed 100% survival in the treated mice, which is superior to IMM40H or IMM01 alone, suggesting a synergistic effect between CD47- and CD70-targeted therapy.

We further evaluated the antitumor activity of IMM40H *in vivo* via Raji orthotopic xenografts. IMM01 (SIRPα-Fc fusion protein) and IMM40H were tested in the study. IMM01 (0.3 mg/kg), IMM40H (1, 3, 10 mg/kg), and IMM01 (0.3 mg/kg) combined with IMM40H (3 mg/kg) were administered twice weekly via tail vein injection for three consecutive weeks. The antitumor activity confirmed that IMM40H substantially improved the survival time in a dose-dependent manner. Overall, 38% (3/8), 75% (6/8), and 75% (6/8) of mice in the three dosages groups (1, 3, and 10 mg/kg, respectively) survived at the endpoint. While CD47 blocker (IMM01) survived 88% (7/8) of the treated mice at the dose of 0.3 mg/kg, the combination of IMM01 (0.3 mg/kg) with IMM40H (3 mg/kg) survived 100% of the treated mice, suggesting a synergistic effect between CD47- and CD70-targeted therapy (**Figure 8**).

#### 3.5.3 *In vivo* activity against renal carcinoma cell (A498) subcutaneous xenograft model

**Figure 9:**
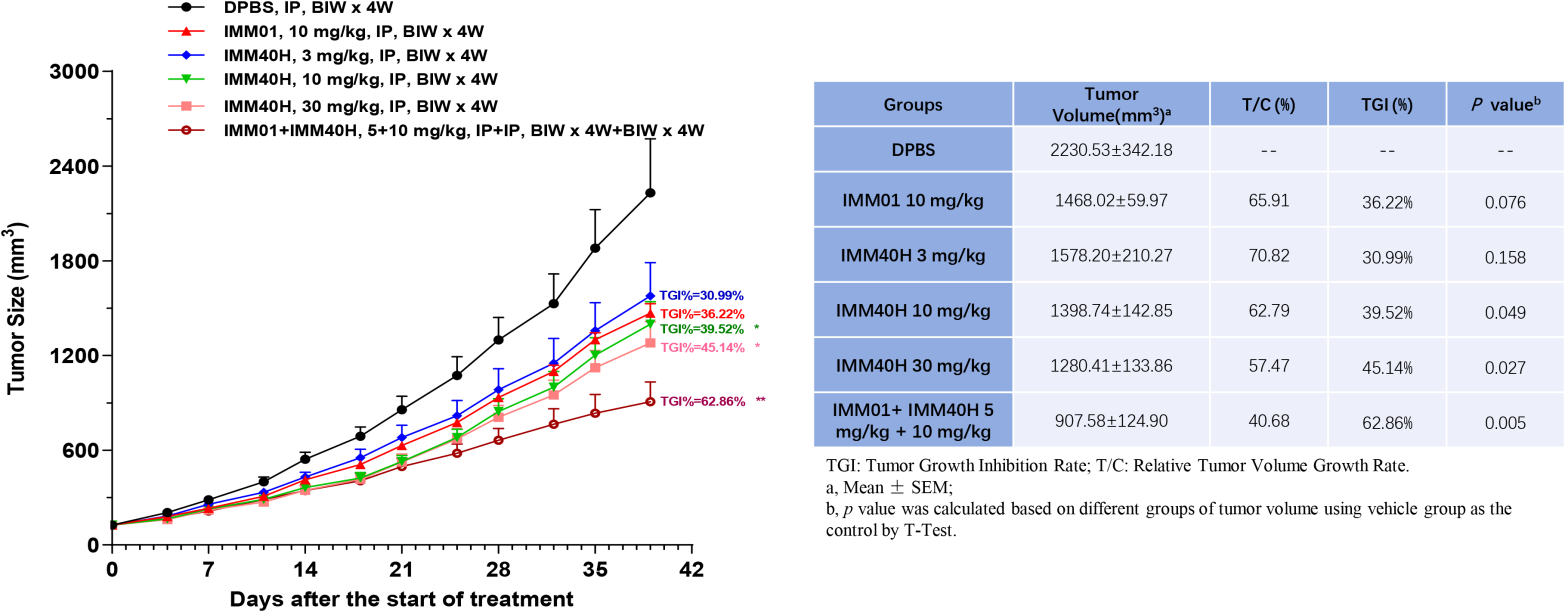
*In vivo* activity against renal carcinoma cell (A498) subcutaneous xenograft model. IMM40H at the doses of 3~30 mg/kg showed a significant tumor inhibition efficiency in a dose-dependent manner. IMM40H demonstrated synergistic effect with CD47-targeted IMM01 (SIRPα-Fc), which is superior to IMM40H or IMM01 alone.

We also performed *in vivo* experiments using an A498 mouse xenograft model to evaluate the antitumor activities of IMM40H combined with IMM01 compared to the activities of IMM40H and IMM01 alone without affecting body weight. IMM01 (10 mg/kg), IMM40H (3, 10, 30 mg/kg), and IMM01 (5 mg/kg) combined with IMM40H (10 mg/kg) were administered twice weekly via intraperitoneal injection for four consecutive weeks. IMM40H at doses of 3–30 mg/kg showed a certain dose-dependent significant tumor inhibition efficacy. IMM40H exhibited a synergistic effect with CD47-targeted IMM01 in treating A498 xenograft renal carcinoma model. Tumor Growth Inhibition (TGI) percentage of Combo (5 + 10 mg/kg), IMM01 (10 mg/kg), and IMM40H (10 mg/kg) was 62.86%, 36.22%, and 39.52%, respectively (**Figure 9**).

### 3.6 IMM40H exhibited a favorable safety and tolerability profile *in vivo*

We used cynomolgus monkeys for pharmacokinetic and toxicological studies. The CD70 amino acid sequence was very similar in human and cynomolgus monkey (86.6%) but only slightly more similar between human and mouse (66.1%). IMM40H can bind to Cyno CD70, whereas mouse CD70 does not. The results of an affinity analysis revealed that IMM40H had an equivalent binding affinity for human and Cyno CD70, but the latter was slightly stronger, as determined by biolayer interferometry (BLI) (**Supplementary Figure 9**). In a GLP-compliant general toxicity study, the potential toxicity of IMM40H was evaluated when it was administered as a single-dose IV infusion to cynomolgus monkeys. All three tested doses of IMM40H (at 20, 50, and 100 mg/kg) were well-tolerated by the monkeys when administered intravenously. At different dosages of IMM40H (up to 100 mg/kg), there were no detectable changes in gross observations, urinalysis, coagulation, serum chemistry, hematological parameters, food intake, body weight, clinical observations, and survival (mortality/morbidity). Thus, IMM40H was well-tolerated at all doses tested, and a single IV infusion of 100 mg/kg was the maximum tolerated dose (MTD) in both male and female cynomolgus monkeys.

**Figure 10:**
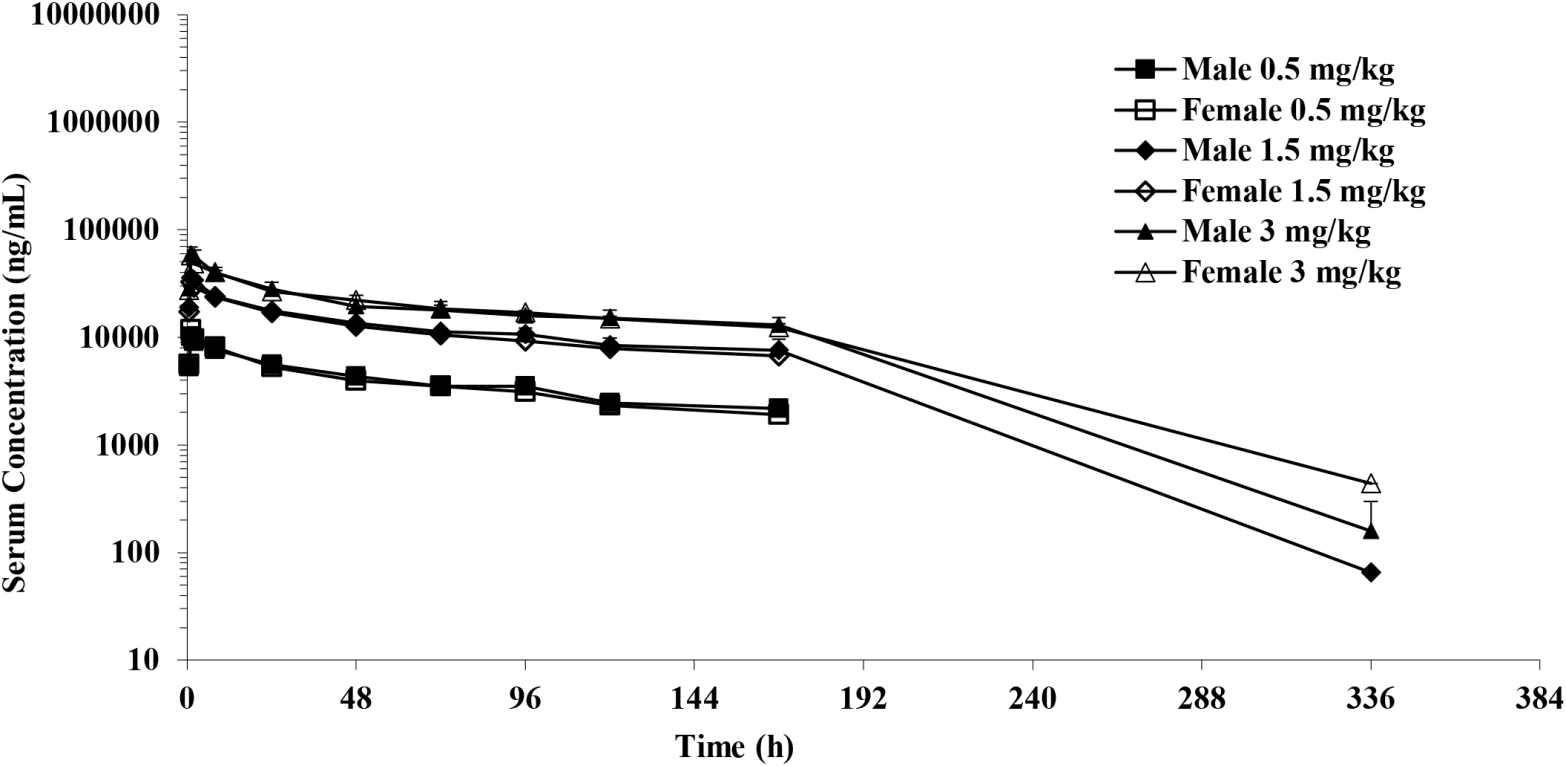
Mean serum concentration after single IV infusion at dose of 0.5,1.5 and 3.0 mg/kg to male and female cynomolgus monkeys.

The PK analysis showed no differences between the sexes at all doses when the area under the serum concentration-time curve was compared from time zero to the last quantifiable concentration (AUC_0-last_) and maximum observed concentration (C_max_) in male and female cynomolgus monkeys. After a single IV infusion of different doses of IMM40H (0.5, 1.5, and 3 mg/kg), the IMM40H showed serum clearance (CL) of 0.00920, 0.00934, and 0.0123 mL/min/kg, respectively, and a half-life (T_1/2_) of 97.3, 75.2, and 48.6 h, respectively. The volume of distribution at steady state (Vdss) was 0.0761, 0.0649, and 0.0711L/kg, respectively. The AUC_0-last_ values were 634000, 2150000, and 3800000 ng·h/mL, respectively. The results showed that the systematic exposure (AUC_0-last_ and C_max_) of IMM40H increased proportionally as the dose increased from 0.5 mg/kg to 3 mg/kg (**Figure 10**, **Table 1**).

**Table 1:** PK Parameters in Cynomolgus Monkeys After Single IV Injection at dose of 0.5, 1.5, and 3 mg/kg of IMM40H.

IMM40H						
Dose Route	IV Infusion		IV Infusion		IV Infusion	
Dose level(mg/kg)	0.5 mg/kg		1.5 mg/kg		3 mg/kg	
PK Parameters	Mean	SD	Mean	SD	Mean	SD
Cmax (ng/mL)	11000	1120	34600	3070	58200	6380
Tmax (h)	1.17	0.408	1.17	0.408	1.00	0.00
T1/2 (h)	97.3	16.8	75.2	50.0	48.6	40.4
Vdss (L/kg)	0.0761	0.00590	0.0649	0.0135	0.0711	0.0112
Cl (mL/min/kg)	0.00920	0.00149	0.00934	0.00223	0.0123	0.00243
AUC0-last (ng•h/mL)	634000	68400	2150000	177000	3800000	440000
AUC0-inf (ng•h/mL)	925000	146000	2820000	747000	4220000	1000000

AUC0-inf, area under the curve to infinite time; AUClast, area under the serum concentration−time curve from time zero to the last quantifiable concentration; Cl, clearance; Cmax, maximum observed concentration; IV, intravenous; PK, pharmacokinetics; T1/2, half-life; Tmax, time of maximum observed concentration; Vdss, volume of distribution at steady state.

We further evaluated the potential toxicity of IMM40H to cynomolgus monkeys when it was administered by IV infusion once a week for five weeks. We also assessed the reversibility, persistence, and delayed occurrence of toxicity following a 42-day recovery period. We found that the administration of IMM40H resulted in IMM40H-related adverse but reversible microscopic changes consisting of mild mononuclear inflammation in the pulmonary interstitium and minimal or mild glomerulonephritis at ≥ 10 mg/kg/dose. No IMM40H-related abnormalities occurred in clinical observations, food consumption, body weight, ophthalmology, body temperature, safety pharmacology (electrocardiogram, blood pressure, respiratory and neurological examinations), urinalysis, immunophenotyping, and macroscopic observations at all doses. Therefore, the highest non-severely toxic dose (HNSTD) of IMM40H was considered to be 30 mg/kg. At this dose, C_max_ and AUC_0–169 h_ of IMM40H were 959,000 ng/mL and 66,400,000 h*ng/mL, respectively, in females, and 877,000 ng/mL and 57,500,000 h*ng/mL, respectively, in males on Day 22 (**Table 2**).

**Table 2:** TK Parameters in Cynomolgus Monkeys After Repeat-dose IV Infusion at dose of 3, 10, and 30 mg/kg of IMM40H.

Dose (mg/kg)	Study Day	Sex	Cmax (ng/mL)	Tmax (h)	AUC0-169h (h*ng/mL)
3	1	Male	63400 ± 16000	1.0 (1.0 - 1.0)	3970000 ± 390000
		Female	72700 ± 4240	1.0 (1.0 - 1.0)	4100000 ± 187000
	22	Male	58700 ± 29300	1.0 (1.0 - 1.5)	1610000 ± 1490000
		Female	81500 ± 20200	1.0 (1.0 - 1.5)	3720000 ± 3280000
10	1	Male	198000 ± 19400	1.0 (1.0 - 1.0)	11600000 ± 1640000
		Female	216000 ± 20300	1.0 (1.0 - 1.5)	13200000 ± 878000
	22	Male	209000 ± 72900	1.0 (1.0 - 1.5)	9810000 ± 10400000
		Female	300000 ± 79300	1.0 (1.0 - 1.5)	21400000 ± 12000000
30	1	Male	614000 ± 55200	1.0 (1.0 - 1.5)	37200000 ± 3780000
		Female	690000 ± 107000	1.0 (1.0 - 1.5)	38200000 ± 4450000
	22	Male	877000 ± 146000	1.0 (1.0 - 1.0)	57500000 ± 15200000
		Female	959000 ± 263000	1.0 (1.0 - 3.0)	66400000 ± 39500000

Data are presented as mean ± SD for Cmax and AUC0-169h values, and median (range) for Tmax. AUC0-169 = area under the serum concentration-time curve (AUC) from time zero to 168 hours post end of infusion (169 hours post start of the infusion, AUC0−169h); Cmax, maximum observed concentration; Tmax, time of maximum observed concentration.

## 4 Discussion

Cancer immunotherapy advanced considerably following the development of therapeutic antibodies that target critical immune checkpoints. In 2011, the U.S. Food and Drug Administration (FDA) authorized the use of ipilimumab for the treatment of melanoma that has spread to other parts of the body (19). Individuals suffering from unresectable or advanced melanoma who do not respond to prior therapy were provided approval to receive treatment by pembrolizumab on September 4, 2014 (20). Nivolumab was granted FDA approval on December 22, 2014, for the treatment of individuals with metastatic or unresectable melanoma whose illness progressed even after ipilimumab therapy, and in patients who tested positive for a BRAF V600 mutation following treatment with a BRAF inhibitor (21). Immune checkpoint blockers are a promising new option for treating malignancies that are beyond the scope of traditional treatment. Patient response rates are still low in most cases, indicating further or combinatorial targeting of immune checkpoints is needed.

Many different types of cancer, both hematologic and solid, have been linked to abnormal expression of CD70 and its receptor CD27. Tumor progression and immunosuppression are linked to the dysregulation of the CD70-CD27 axis in the tumor and its microenvironment (22). Since CD70 is normally expressed only by a small percentage of cells in the lymphoid compartment, therapies that specifically target this protein should have few unintended consequences. When tumor cells overexpress CD70, CD27 expression in tumor-infiltrating Tregs may facilitate immune evasion. Many different types of cancer, including renal cell carcinoma, glioblastoma, thymic carcinoma, nasopharyngeal carcinoma, T-anaplastic large-cell lymphoma, Waldenström’s macroglobulinemia, and Hodgkin and non-Hodgkin lymphomas, may respond well to therapies that target CD70 (13, 15, 23–28).

Monoclonal antibodies, antibody-drug conjugates (ADCs), and CAR-T-based treatments have shown that CD70 is the optimal target due to its very limited expression pattern in certain blood cancers and solid tumors. Several drugs based on mAbs (SEA-CD70, MDX1411, and cusatuzumab), ADCs (ARX305, MDX-1203, AMG172, SNG-CD70, and SNG-75), and CAR-Ts (CTX130, CD70–001, ALLO-316, and GIMIIRB-20006) are currently being tested in clinical trials for the treatment of CD70-related diseases (29). Combining cisplatin and docetaxel with anti-CD70 treatment (Cusatuzumab) can boost antitumor immune responses in NSCLC patients, as shown by preclinical evidence (30). Defucosylated anti-CD70 monoclonal antibody cusatuzumab (ARGX-110) prevents tumor immune escape by blocking the survival of Tregs and restoring normal myeloid differentiation. Phase I dose-escalation study of cusatuzumab showed good tolerability, pharmacokinetics, and preliminary antitumor activity at all dose levels (0.1, 1, 2, 5, and 10 mg/kg) in patients with advanced CD70-positive malignancies (31). Cusatuzumab also inhibits LSC proliferation, reduces leukemic blast cells, and blocks CD70/CD27 signaling (8, 9). Patients with previously untreated AML who were ineligible for intense chemotherapy responded well to a combination of cusatuzumab and azacitidine, as determined by a Phase I/II study (10–12).

We obtained IMM40H through hybridoma screening and antibody humanization techniques. IMM40H specifically binds to the CD70 target and has higher affinity and stronger blocking activities compared to competitor anti-CD70 antibodies, including Cusatuzumab. IMM40H can interrupt the proliferation and activation of Treg cells by inhibiting CD70/CD27 signaling. Additionally, IMM40H also showed potent Fc-dependent effector functions (ADCC/CDC/ADCP) via S298A, E333A, and K334A substitution in the Fc region, resulting in a strong immune attack on hematologic malignancies and potent therapeutic efficacy. Our preclinical data also suggested that IMM40H has potent antitumor activity in the U266B1 multiple myeloma tumor model, eradicating subcutaneously established tumors even at a dose as low as 0.3 mg/kg. Moreover, IMM40H (0.3 mg/kg) showed a therapeutic effect faster than cusatuzumab (1 mg/kg). With cusatuzumab (1 mg/kg), tumors were cleared in five of the six mice. A strong synergistic effect of IMM01 (SIRPα-Fc fusion protein) and IMM40H was found on Burkitt’s lymphoma Raji and renal carcinoma cell A498 tumor models. Synergistic antitumor activity between CD47-and CD70-targeted therapy acts as a foundation for their combined application in future clinical studies. IMM40H has a favorable safety and tolerability profile, considering that significant IMM40H-related toxic side effects were not observed. The HNSTD for repeat-dose toxicity and MTD for single-dose toxicity were up to 30 mg/kg and 100 mg/kg, respectively, in cynomolgus monkeys. We obtained IND approval for IMM40H from the NMPA and the FDA in August 2022, and we aim to initiate Phase I clinical studies.

## 5 Conclusions

Anti-CD70 targeted combinatorial therapy was effective in preclinical and clinical investigations. Although AML is the primary indication of monotherapy and combinatorial therapy, this strategy might be applied to other tumor types also. The CD70-CD27 axis was studied extensively for its role in tumor promotion and immune evasion in cancer, and new insights into its putative molecular processes emerged. Therefore, methods to suppress the signaling pathways implicated in the CD70-CD27 axis might offer attractive new therapeutic possibilities along with current techniques that rely on targeting CD70. Pre-clinical findings showed that IMM40H has a higher binding ability, stronger ability to block the interaction of CD70/CD27, and stronger ability to kill tumor cells than other CD70 competitors. We aim to investigate the safety and efficacy of IMM40H in clinical trials for hematomas and solid tumors. It might emerge as a novel, safe, and effective therapeutic option for treating cancers.

## Data availability statement

The original contributions presented in the study are included in the article/**Supplementary Material**. Further inquiries can be directed to the corresponding author.

## Ethics statement

The animal study was approved by the Institutional Animal Care and Use Committee (IACUC) of WuXi AppTec and Medicilon Biology. The study was conducted in accordance with the local legislation and institutional requirements.

## Author contributions

WT designed and directed the study. WT and SL wrote the manuscript draft. SL and DC carried out the experiments. SL, DC, HG, DL, CY, RZ, TX, FZ, XB, YY, NS, WZ, LZ, GZ, LP, XT performed the experiments. All authors critically reviewed and approved the final manuscript.
