# Supplementary material for: The novel high-affinity humanized antibody IMM40H targets CD70, eliminates tumors via Fc-mediated effector functions, and interrupts CD70/CD27 signaling
Source: Front Oncol. 2023 Oct 2;13:1240061. doi: 10.3389/fonc.2023.1240061 (PMC10578964; doi:10.3389/fonc.2023.1240061)
Supplement: Supplementary file 1 [file DataSheet_1.docx]

Supplementary Material

The Novel High-Affinity Humanized Antibody IMM40H Targets CD70, Eliminates Tumors Via Fc-mediated Effector Functions, and Interrupts CD70/CD27 Signaling

Song Li^1#^, Dianze Chen^1#^, Huiqin Guo^1^, Dandan Liu^1^, Chunmei Yang^1^, Ruliang Zhang^1^, Tianxiang Wang^1^, Fan Zhang^1^, Xing Bai^1^, Yanan Yang^1^, Nana Sun^1^, Wei Zhang^1^, Li Zhang^1^, Gui Zhao^1^, Liang Peng^1^, Wenzhi Tian^1*^

*** Correspondence:**Wenzhi Tian
wenzhi.tian@immuneonco.com

# Supplementary Figures


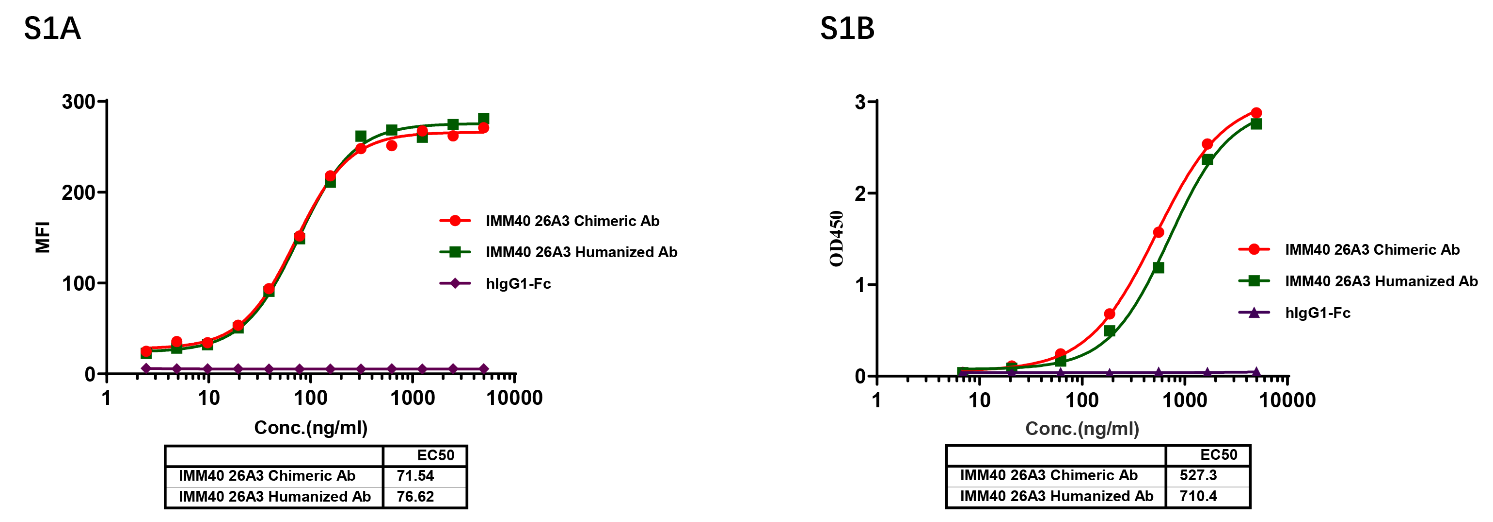


**Supplementary Figure 1.** Binding of the humanized anti-CD70 antibody IMM40H and its chimeric version to U266B1 cells, determined by flow cytometry assays (2A), to human trimer CD70 protein (2B), determined by ELISA. The humanized CD70 antibody has equivalent CD70-binding activity to the chimeric antibody.


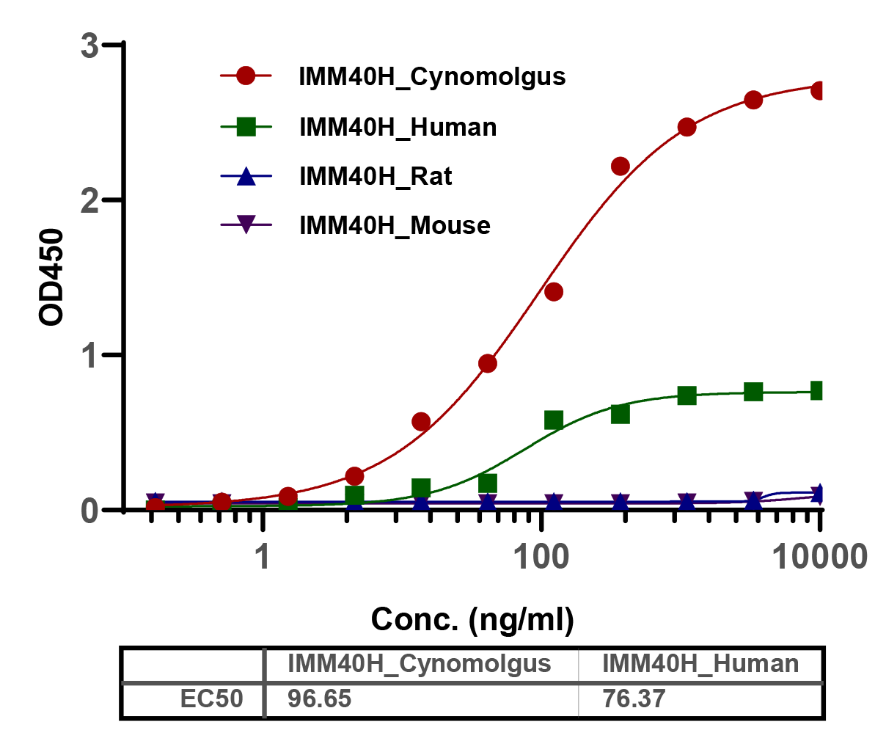


**Supplementary Figure 2.** CD70 target species cross-reactivity was analyzed by ELISA. IMM40H can bind to the CD70 protein of human and cynomolgus monkey, but not of mouse and rat.


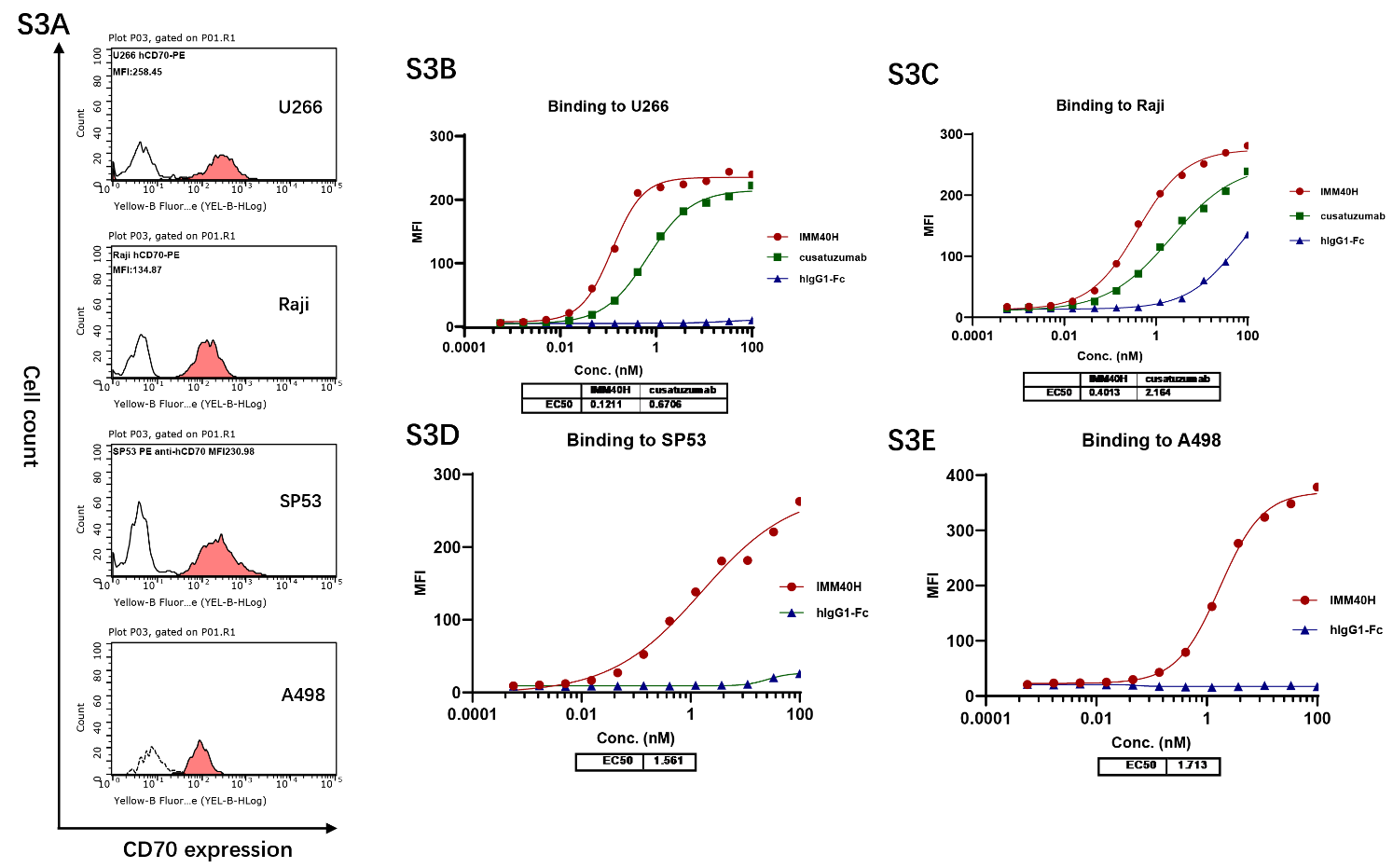


**Supplementary Figure** **3.** The binding of IMM40H to CD70+ tumor cells was evaluated by FACS. A. Detection of CD70 expression in different tumor cells. B. The binding of IMM40H to multiple myeloma U266B1 cells. C. The binding of IMM40H to Burkitt’s lymphoma Raji cells. D. The binding of IMM40H to mantle cell lymphoma SP53. E. The binding of IMM40H to renal cell carcinoma A498. The binding of IMM40H to U266, Raji, A498, and SP53 cells with high affinity in the low-nanomolar range.


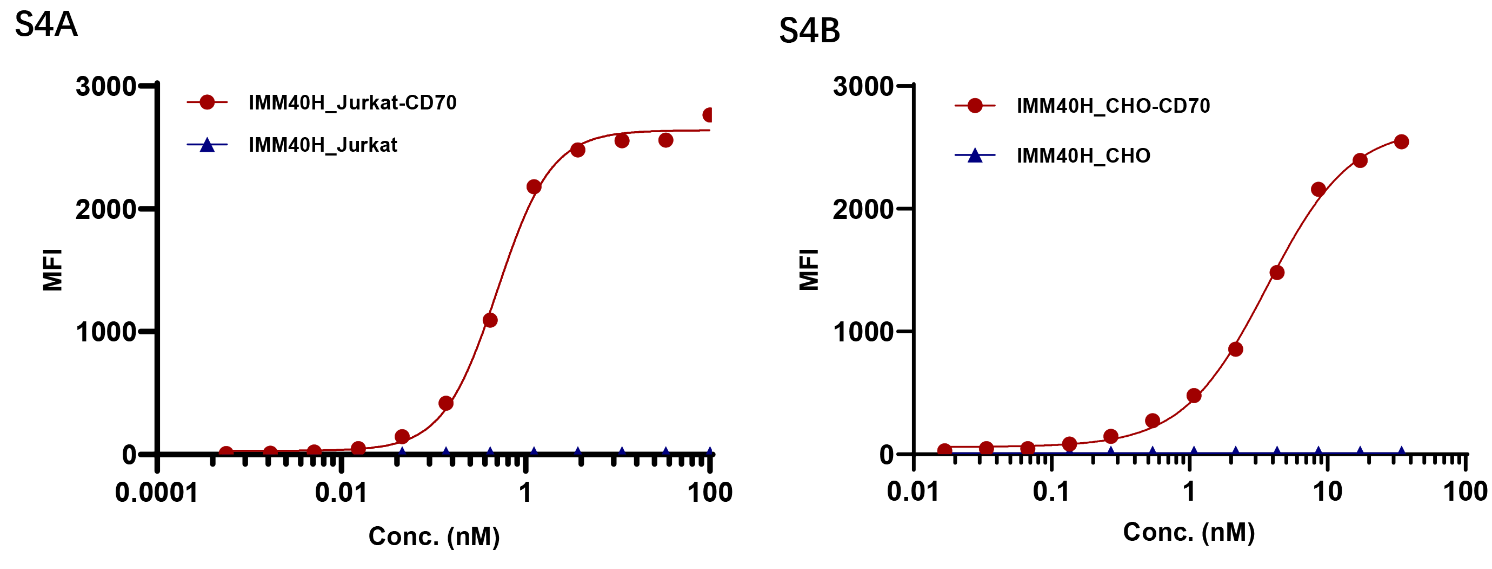


**Supplementary Figure 4.** IMM40H specifically bound to the CD70 target. A. The binding of IMM40H to Jurkat-CD70 cell. B. The binding of IMM40H to CHO-CD70 cells. IMM40H did not bind to CD70-negative Jurkat and CHO cells, but it bound after transfection with CD70, demonstrating its specificity for CD70.


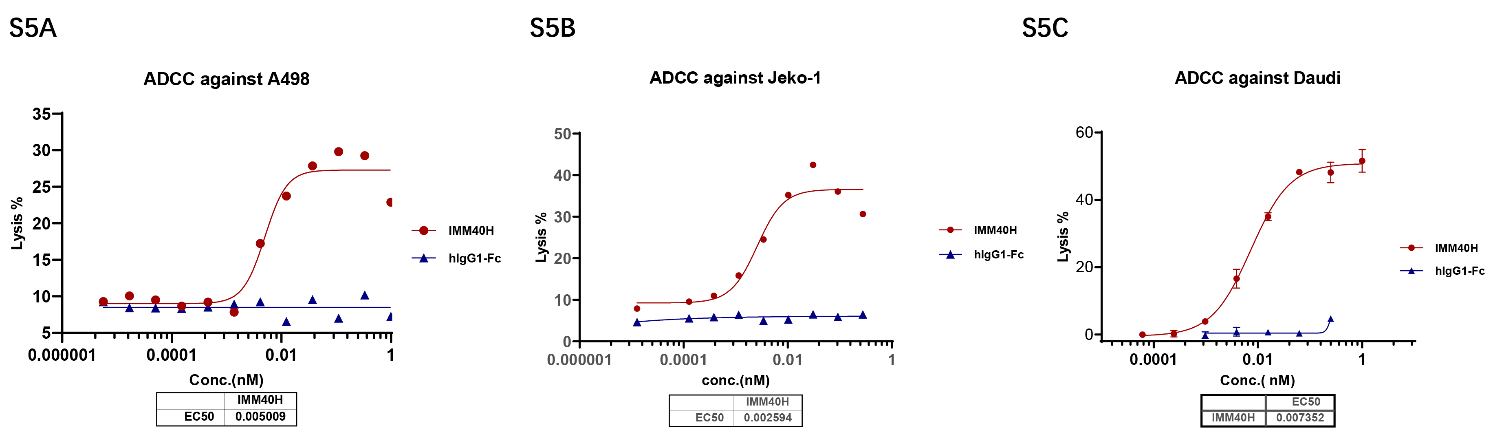


**Supplementary Figure 5.** The ADCC activity against CD70+ tumor cells was measured using FcR-TANK. A. IMM40H induced ADCC against A498. B. IMM40H induced ADCC against Jeko-1. C. IMM40H induced ADCC against Daudi.


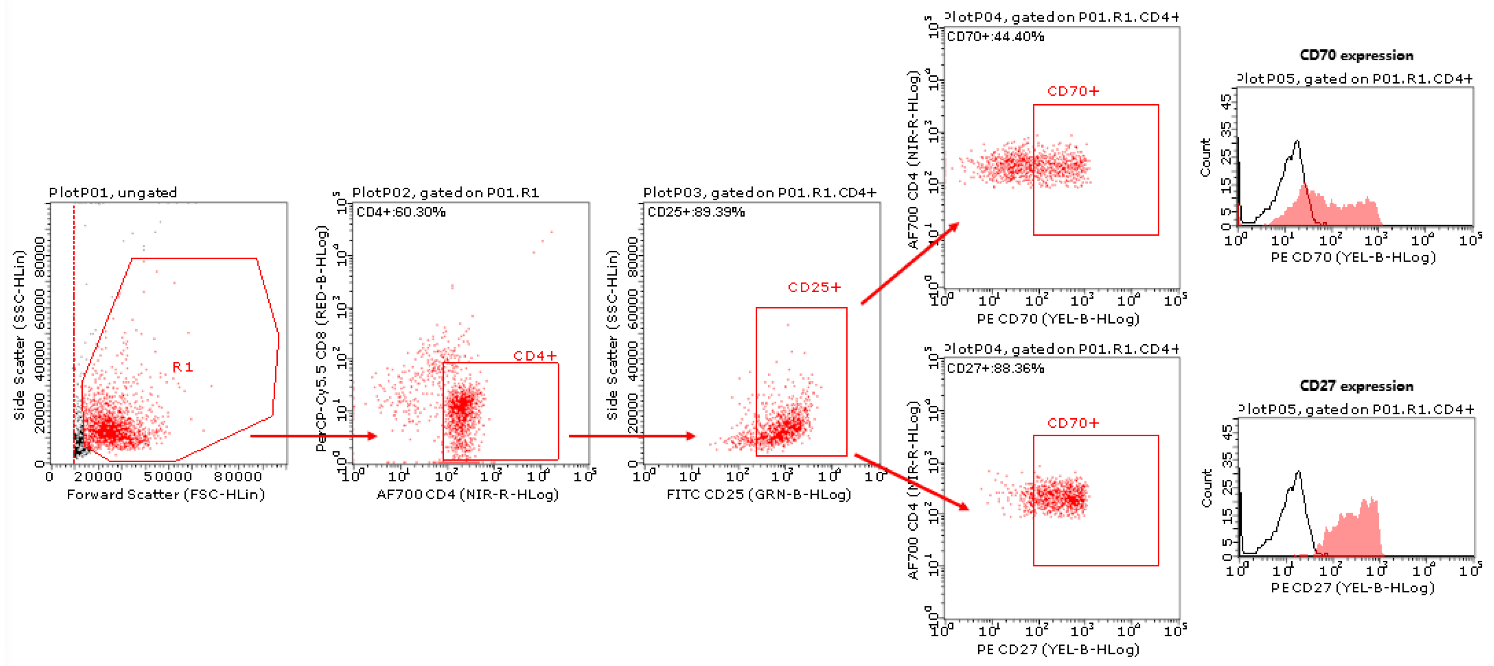


**Supplementary Figure 6.** Analysis of CD27 and CD70 expression in activated Tregs. Treg cells (Sailybio, Cat#XFB-nTreg-02BA) were identified using CD4 (BD biosciences, Cat#557922) and CD25 (BD biosciences, Cat#302604) markers. The expression of CD27 and CD70 were analyzed using fluorescent labeled antibodies against CD27 (Biolegend, Cat#302808) and CD70 (Biolegend, Cat#355104), respectively. The results showed that Tregs expressed low levels of CD27 and CD70.


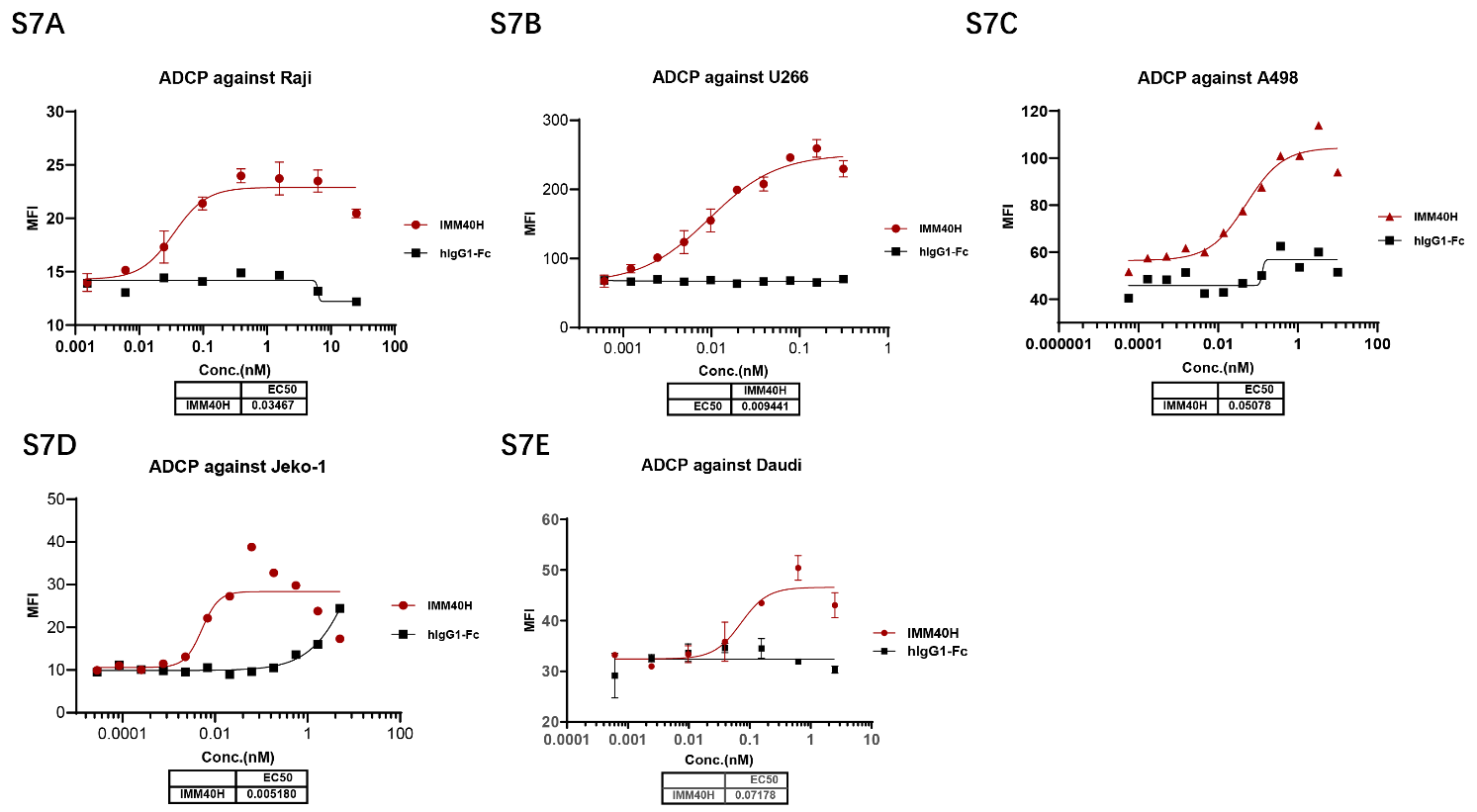


**Supplementary Figure 7.** The ADCP activity against CD70+ tumor cells were measured using THP-1 cell. A. IMM40H induced ADCP against Raji. B. IMM40H induced ADCP against U266. C. IMM40H induced ADCP against A498. D. IMM40H induced ADCP against Jeko-1. E. IMM40H induced ADCP against Daudi. MFI represents the fluorescence intensity of macrophages after phagocytosis of labeled target cells. IMM40H induces potent ADCP against CD70+ tumor cells.


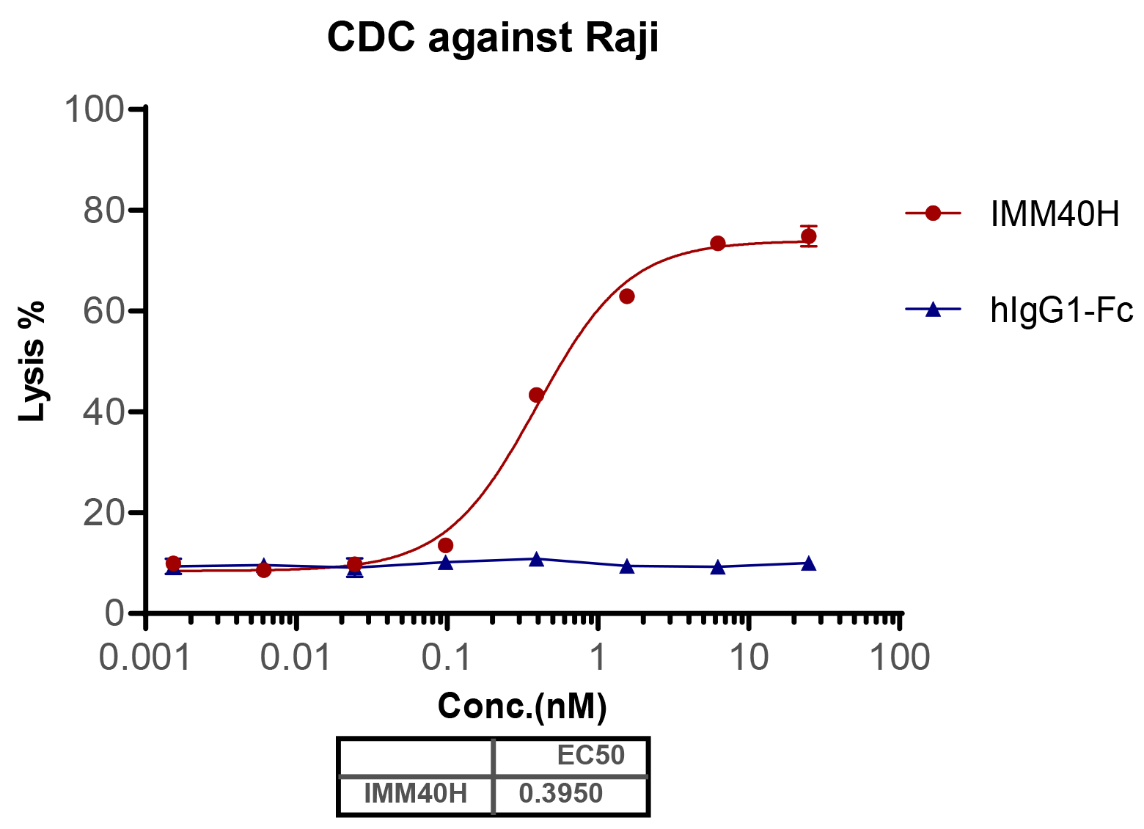


**Supplementary Figure 8.** The CDC activity against Raji was measured using human serum complement. The lysis EC_50_ for IMM40H was 0.395 nM in Raji, however, IMM40H had no CDC activity against other CD70+ tumor cell lines, including U266, Daudi, Jeko-1, or A498 (data not presented).


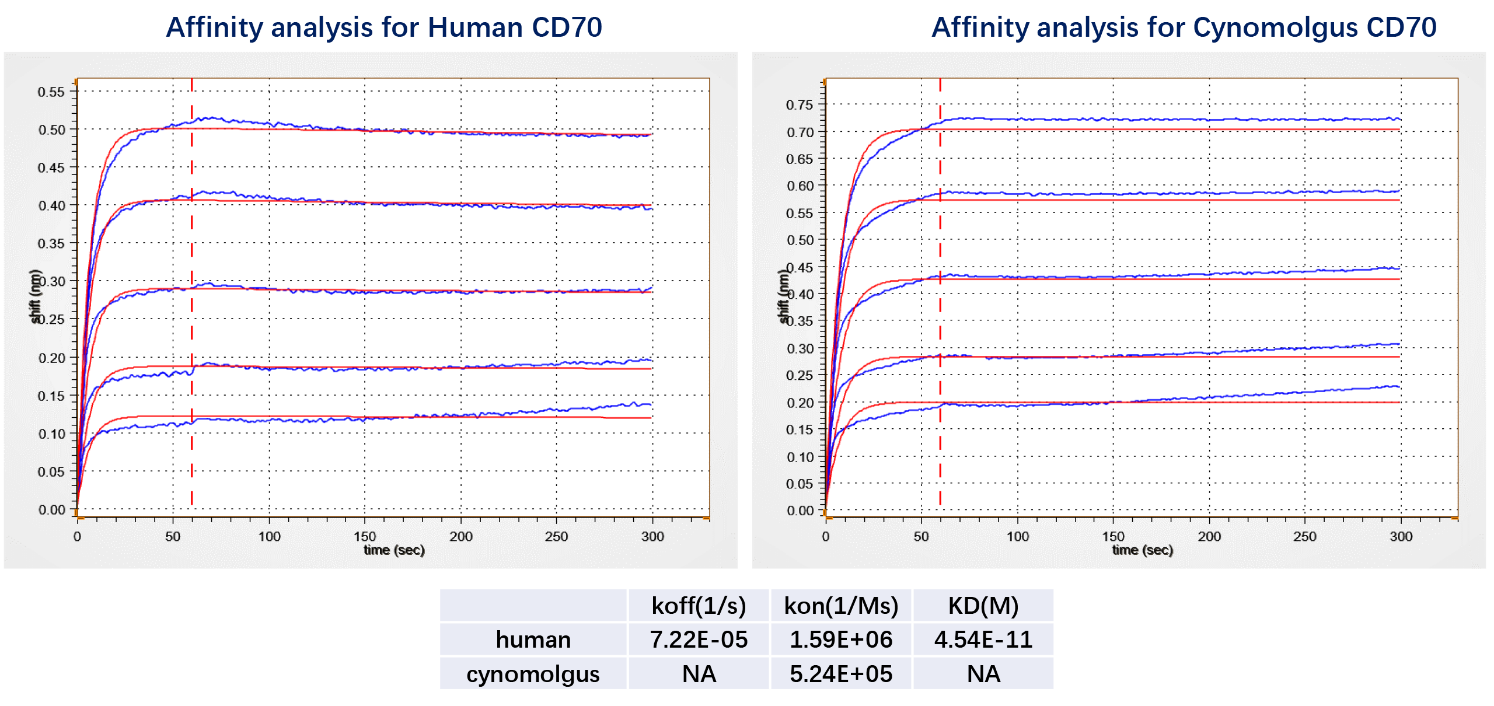


**Supplementary Figure 9.** Affinity analysis of IMM40H to human and cynomolgus CD70 by BLI. Affinity analysis revealed that IMM40H has equivalent binding affinity for human and Cyno CD70, with the latter is slightly stronger.
